# Investigating the Spectral Characteristics of High-Temperature Gases in Low-Carbon Chemical Pool Fires and Developing a Spectral Model

**DOI:** 10.3390/toxics13100877

**Published:** 2025-10-14

**Authors:** Gengfeng Jiang, Zhili Chen, Yaquan Liang, Peng Li, Qiang Liu, Lv Zhou

**Affiliations:** 1 Guangxi Key Laboratory of Environmental Pollution Control Theory and Technology, Guilin University of Technology, Guilin 541006, China; jianggf@glut.edu.cn; 2 College of Environmental Science and Engineering, Guilin University of Technology, Guilin 541004, China

**Keywords:** low-carbon chemicals, chemical fires, high-temperature pollutant gases, characteristic spectra, spectral radiation modeling

## Abstract

Low-carbon chemical fires pose significant hazards, and remote sensing of high-temperature gas emissions from these fires is a critical method for identifying and assessing their environmental impact. Analyzing the spectral characteristics of gases produced by low-carbon chemical pool fires and developing spectral radiation models can establish a foundation for remote pollution monitoring. However, such studies remain scarce. Using a custom-built high-temperature gas spectroscopy platform, this study extracts spectral features of gases emitted by low-carbon chemical pool fires. We investigate spectral interference mechanisms among combustion products and develop a high-precision spectral radiation model to support remote fire pollution monitoring. Experimental results reveal distinct spectral bands for key gases: CO_2_ peaks near 2.7 μm and 4.35 μm, SO_2_ at 4.05 μm, 7.5 μm, and 9.0 μm, NO at 5.5 μm, and NO_2_ at 3.6 μm and 6.3 μm. The proposed spectral radiation model accurately simulates the position and shape of spectral peaks. For carbon disulfide and acetonitrile combustion products, the model achieves prediction accuracies of 83.4–96.9% and 79.2–95.3%, respectively.

## 1. Introduction

Natural and human-made disasters persistently threaten ecological balance and societal stability. Among these, fires pose a severe and underestimated risk. Annual statistics reveal staggering human casualties and economic losses from fires, particularly in the chemical industry, where their impact is amplified [1,2].

Low-carbon chemicals are defined as substances produced from greenhouse gases (e.g., CO_2_, N_2_O, HFCs, PFCs, SF_6_) or through processes that substantially reduce emissions. Their development is critical for advancing low-carbon economies and is now a priority for industries aiming to minimize their carbon footprints. However, flammable low-carbon chemicals present significant hazards during production, transportation, and storage, with risks of explosions and potential misuse in attacks. Recent incidents have caused severe economic, human, and environmental damage, underscoring their societal risks [3,4]. Aerospace remote sensing offers a rapid, large-scale solution for monitoring pollution from low-carbon chemical fires. Identifying spectral signatures of these fires is essential for improving remote detection capabilities.

The spectral characteristics of chemical combustion serve as critical indicators for detecting and identifying pollution from chemical fires. Flame spectra primarily originate from soot radiation and molecular emissions of H_2_O and CO_2_ in combustion products [5]. Low-carbon chemical flames exhibit distinct spectral bands at 1.87 μm and 2.7 μm for H_2_O, and 2.7 μm and 4.3 μm for CO_2_. Leading institutions have developed comprehensive spectral databases, including NIST’s Chemistry WebBook and AIST’s SDBS (Spectral Database for Organic Compounds), which provide extensive reference data for combustion analysis [6,7]. The GEISA spectral line parameter database covers 58 molecules (145 isotopic variants) with over 6.7 million entries spanning 10^−6^ to 35,877 cm^−1^. The HITRAN database from AFGL contains high-resolution spectral data for 49 atmospheric components and pollutants, serving as essential references for quantitative gas analysis [8,9,10,11,12,13,14,15]. The HITEMP database specializes in high-temperature gas spectroscopy, showing improved accuracy for H_2_O, CO_2_, CO, NO, and OH through recent updates. However, uncertainties persist in some parameters [16,17,18,19]. For instance, CO_2_ spectral parameters in HITRAN and HITEMP databases show measurement uncertainties exceeding 20% for most line intensities, with only minor exceptions below 20% [20]. The applicability of these spectral databases for pollutant identification under fire conditions requires further validation. Current research predominantly focuses on common combustion products (H_2_O and CO_2_), while spectral analyses of characteristic pollutants like NOx and SOx remain limited. The interference mechanisms between combustion products also require deeper investigation.

Current research on the spectral characteristics of low-carbon chemical combustion focuses on two approaches: (1) Radiation heat transfer modeling using spectral data [21,22,23,24,25,26,27,28]. For instance, the RADCAL software V3.8.5 employs narrow-band methods to calculate combustion radiation, while flame spectral data are used to derive parameters like mass burning rates and thermal radiation values. However, these studies lack in-depth analysis of spectral features specific to combustion pollutants, limiting their applicability for pollution detection. (2) Differentiating pollutants via flame spectral signatures [29,30]. Techniques such as wavelet analysis distinguish fuel flames (e.g., oil) from other combustibles (e.g., alcohol, wood, paper), and spectral features combined with GOCI-Rrc satellite imagery identified carbonaceous aerosols as the primary pollutants in the Tianjin Port explosion. In smoke-free fire scenarios, molecular radiation from H_2_O, CO_2_, and CO dominates total fire radiation [31,32]. Recent studies highlight significant differences in infrared spectral properties of combustion-derived pollutants, enhancing remote sensing capabilities for fire pollution detection [33,34]. For example, Parent observed prominent absorption bands in atmospheric low-temperature gases within the 200–2200 cm^−1^ range, likely caused by high-temperature CO_2_ emissions during combustion [35]. Bordbar demonstrated that optimized filter responses amplify fire-induced spectral signals by 49–514% compared to blackbody emitters [36,37,38,39,40,41]. Challenges remain, however, due to spectral overlaps in certain chemical flames. Qiao visualized tail flame structures in rocket engines, identifying H_2_O and CO_2_ emission bands—consistent with findings from LNG flame studies [37]. Additionally, molecular similarities among combustion products create spectral interference effects [42,43,44,45].

Current research predominantly focuses on the spectral radiation properties of known chemical combustion processes, with limited attention to low-carbon chemical fire pollution from an aerospace remote sensing perspective. The existing satellite bands are concentrated in the range of 0.443–2.20 μm and 10.9–12.0 μm. The characteristic bands of SO_2_ (7.5 μm) and NO (5.5 μm) identified in this study can fill the monitoring blind area of satellites in the 2.2–10.9 μm range, realize the direct identification of characteristic pollutants from low-carbon chemical fires, and avoid the high error of traditional satellites relying on the indirect inversion of CO_2_. Meanwhile, the laboratory spectral resolution is as high as 0.12 cm^−1^ with an error of less than 15%, which can provide ground truth values for satellite low-resolution data (e.g., 0.35 cm^−1^ for Sentinel-2 MSI and 3 cm^−1^ for MODIS). It is worth mentioning that the “dual-band combination logic” proposed in this paper can help satellites distinguish between traditional hydrocarbon fires and low-carbon chemical fires.

There are significant differences in spectral characteristics between low-carbon chemical fires and traditional hydrocarbon fuel fires (such as those of petroleum and natural gas). Most studies emphasize common combustion byproducts such as H_2_O, CO_2_, and soot, while neglecting spectral analyses of characteristic pollutants like NOx and SOx. This gap limits the applicability of existing findings for detecting and distinguishing specific pollutants from diverse chemical fires. The combustion of low-carbon chemicals (e.g., CS_2_, acetonitrile) releases sulfur-containing/nitrogen-containing pollutants such as SO_2_ (7.5 μm) and NO (5.5 μm), which can serve as exclusive identification markers. In contrast, traditional hydrocarbon fires only rely on the common bands of CO_2_ and H_2_O. In low-carbon chemical fires, CO_2_ and SO_2_ have overlapping bands in the 4.3–4.6 μm and 7–10 μm ranges, and the high-temperature decomposition of NO_2_ dynamically changes the spectral characteristics. However, the interference in traditional hydrocarbon fires only comes from the slight overlap between H_2_O and CO_2_, making the analysis less difficult than that for low-carbon chemicals. It should be noted that existing studies only mention the spectral overlap between NOx/SOx and CO_2_, but do not quantify the degree of interference at high temperatures (e.g., 1023 K) and the effectiveness of deconvolution methods (such as NNLS), which cannot support the collaborative identification of multiple components.

In summary, to address this, our study employed a custom-built high-temperature gas spectral testing platform to extract spectral signatures of gas-phase products from low-carbon chemical pool fires. We established a stable high-temperature combustion environment, investigated spectral interference mechanisms among combustion products, and developed a high-precision spectral radiation model. These advancements provide a foundational framework for remote sensing technologies to identify and monitor low-carbon chemical fire pollutants.

## 2. Materials and Methods

### 2.1. High-Temperature Gas Spectral Testing Platform

**Figure 1:**
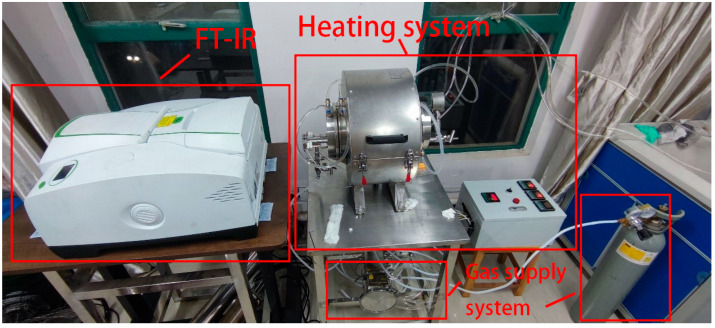
High-Temperature Gas Spectral Testing Platform.

Low-carbon chemical pool fires exhibit significant turbulent behavior, leading to substantial fluctuations in combustion product concentrations, temperatures, and spectral radiation. These variations hinder accurate measurements of flame field parameters such as temperature, concentration, and spectral data. To address this, a stable high-temperature gas environment was essential. This study developed a custom spectral testing platform (the platform is illustrated in Figure 1) to generate controlled high-temperature combustion conditions. Experiments were conducted in an enclosed chamber to measure spectral radiation from both individual and mixed gas-phase combustion products under stable conditions, providing critical spectral signature data for model development. The setup utilized a Fourier-transform infrared spectrometer, Bruker Optik GmbH, Ettlingen, Germany and an HFY-203D blackbody radiator, Shanghai Fuyuan Optoelectronics Technology Co., Ltd., Shanghai, China.

The emissivity of the HFY-203D blackbody is 0.995 (in compliance with JJG 856-2015 “Calibration Specification for Blackbody Radiation Sources” [46]). This blackbody is used for both lens transmittance measurement and gas spectral calibration: the blackbody radiation spectrum is collected at 800 K to establish a linear relationship between “radiance–spectral signal”, ensuring that the unit of gas spectral radiance is an absolute unit (W/(cm^2^·μm·sr)).

The Fourier Transform Infrared Spectrometer (FT-IR) produced by PerkinElmer (Shelton, CT, USA) (see Table 1 for instrument parameters) uses the Blackman–Harris apodization function. The spectral bands with high instrument noise in the range of 350–600 cm^−1^ are discarded. During the test, the spectral wavenumber range is 700–8000 cm^−1^, the spectral resolution is 4 cm^−1^, the cumulative number of scans is 2, the scan type is set to single-beam, and a high-precision liquid nitrogen-cooled MCT detector is used.

To minimize background radiation interference from daylight, all experiments were conducted at night under sunlight-free conditions. The platform consists of four independent modules: heating, spectral data acquisition, gas distribution, and water cooling. Connected via standard connectors, it can be disassembled for transportation and on-site assembly outdoors. The spectrometer can perform remote data collection via a 10 m optical fiber to avoid high-temperature interference. The water cooling system can be replaced with an air cooling module (the cooling efficiency is reduced to 80% of that of water cooling, but the weight is reduced by 60%), which is suitable for outdoor scenarios without water sources; after modification, the cost of a single outdoor experiment is approximately 30% higher than that in the laboratory, but it enables in situ spectral collection of fires involving CS_2_, acetonitrile, etc.

During platform assembly, we observed that the water-cooled flange provided limited cooling to the lens. When the gas in the chamber was heated to 800 °C, the monocrystalline silicon lens temperature reached 200 °C, causing significant changes in lens transmittance and inherent radiation spectra, which compromised spectral measurement accuracy. To address this, we quantified the interference effects of lens transmittance and self-emission on experimental results. To maintain a constant radiative background during high-temperature lens transmittance measurements, the HFY-203D blackbody furnace was stabilized at 800 °C. This temperature was selected based on the furnace’s stable radiative brightness and minimal thermal fluctuations at 800 °C. The lens transmittance is calculated using Equation (1).

(1) $$\tau \left(v\right)=\left(E_{2}+E_{3}\right)/E_{1}$$

$\tau \left(v\right)$ is the lens transmittance at varying wavenumbers, *E*_1_ denotes the blackbody radiative energy measured through the chamber, *E*_2_ represents the radiative energy measured through the chamber after inserting the lens, and *E*_3_ corresponds to the lens’s inherent radiative energy.

The measurement process for E_1_, E_2_, and E_3_ is as follows:

(1) Measurement process of E_1_: Turn on the blackbody furnace and slowly heat it up at a temperature gradient of 100 °C. When the temperature reaches the target temperature of 800 °C, keep it constant. Then, open the valve at the air inlet and then open the valve at the air outlet of the gas distribution system to introduce inert gas and exhaust the air inside cavity 2. Nitrogen is selected as the background gas in this experiment. Move the rotating chassis to position the heating chamber in front of the blackbody and Fourier transform infrared (FT-IR) spectrometer. By adjusting the angle of the rotating chassis and its position on the slide rail, ensure that the radiation from the blackbody can pass through the chamber and fully enter the field of view of the FT-IR spectrometer. After completing the above operations, turn on the FT-IR spectrometer to scan and obtain E_1_.
(2) Measurement process of E_2_: Maintain the position of each part on the platform during the measurement process of E_1_. Gently pull the handle to open the lens heating chamber, place the lens to be tested inside, adjust the sliding block, and press the lens firmly against the high-temperature heating plate to ensure uniform heating. Then, open the valve at the air inlet, and then open the valve at the air outlet of the gas distribution system to introduce inert gas or nitrogen to exhaust the air inside the cavity. Turn on the lens heating temperature control box to raise the lens to the target temperature. After completing the above operations, use an FT-IR spectrometer to scan and obtain E_2_.
(3) Measurement process of E_3_: Move the blackbody furnace to the other side of the slide rail to ensure that it is not within the field of view of the FT-IR spectrometer, and there is no light source within the field of view. Rotate the rotating chassis by 180°, placing the lens end within the field of view of the FT-IR spectrometer. After completing the above operations, use the FT-IR spectrometer to scan and obtain E_3_.

There is a certain degree of uncertainty in E_3_ (lens radiant energy). This is because key parameters have an impact on accuracy: temperature (+0.42%/K, increasing to +0.65%/K at high temperatures) > incident angle (−1.8%/°) > transmittance fitting error (±1.5% per 1% error) > surface contamination (−0.9% per 1% decrease in transmittance). This error can be optimized (e.g., by integrating a precise temperature control module, mechanical positioning, segmented fitting, etc.).

### 2.2. Spectral Denoising and Smoothing Processing

During high-temperature gas spectral testing, the monocrystalline silicon lens within the field of view partially absorbs the gas’s spectral radiance *C*_1_(*v*), which is then detected by the FT-IR spectrometer. The total signal entering the spectrometer comprises *C*_1_(*v*) and the lens’s inherent radiation *E*_3_. To derive the true radiative brightness *L*(*v*) of the gas, the lens-induced interference must be eliminated by isolating *C*_*n*_(*v*), the corrected spectral radiance. This correction is achieved using Equation (2):

(2) $$C_{n}\left(v\right)=C_{1}\left(v\right)/\tau \left(v\right)+E_{3}$$

*C*_*n*_(*v*) is the initial spectral radiance measured by the FT-IR spectrometer, *C*_1_(*v*) is the spectral radiation value obtained by interference from high-temperature lenses, *τ*(*v*) denotes the transmittance of the high-temperature lens, *E*_3_ represents the inherent radiative energy of the lens.

In addition to interference from the high-temperature lens, reflected light from components such as the quartz tube, flange, and furnace heating filaments also enters the FT-IR spectrometer. This introduces baseline drift, excessive spectral noise, and spectral peaks which are difficult to distinguish, complicating data interpretation. To mitigate these effects, a background spectrum must first be acquired using a gas with negligible radiative features. Nitrogen, a diatomic molecule, exhibits extremely weak spectral signatures—its radiative brightness at equivalent temperatures is approximately five orders of magnitude lower than that of carbon dioxide [47]. Thus, nitrogen spectra at varying temperatures were selected as the background reference. The correction is calculated using Equation (3):

(3) $$C_{0}\left(v\right)={C_{0}}^{\prime }\left(v\right)-C_{N}\left(v\right)$$

*C*_0_(*v*) is the corrected spectral radiative energy of the target high-temperature gas after interference removal, *C_N_*(*v*) represents the radiative energy of the high-temperature nitrogen gas used for background correction, *C*_0_′(*v*) denotes the uncorrected radiative energy of the gas prior to background subtraction.

Despite background correction, residual baseline drift and spectral noise persist in the high-temperature gas spectra. To enhance the visibility of characteristic spectral peaks, this study employs the AirPLS algorithm—an adaptive iteratively reweighted penalized least squares method (Parameters of the AirPLS algorithm: regularization parameter λ = 10^6^, number of iterations = 50, stopping tolerance = 10^−6^; Whittaker smoothing window size = 15; Gaussian convolution FWHM = 0.1 cm^−1^, and the convolution parameters are set based on the spectral line half-width of the HITRAN 2020 database (Ontar Corporation, North Andover, MA, USA))—to suppress noise and oscillations. This approach preserves the overall spectral trend while sharpening peak resolution. However, this algorithm has certain limitations. When the concentration of a certain component is extremely low (e.g., NO 0.5%) and the signal of its characteristic peak (5.5 μm) is close to the baseline noise (<0.005 W/(cm^2^·μm·sr)), the iterative weight of the algorithm will underestimate the residual of the peak, resulting in a peak area error of 15–20% and a detection rate reduced to 85%. The Whittaker smoothing window (15) causes peak shape broadening for narrow peaks (e.g., NO 5.5 μm) and suppresses edge signals for wide peaks (e.g., SO_2_ 9.0 μm), resulting in an increase in peak height error from ±2.8% for a single gas to ±5.1% for mixed gases. Moreover, this algorithm can only remove baseline noise and cannot distinguish extremely weak overlapping peaks (e.g., CO_2_ 4.35 μm and SO_2_ 4.4 μm). It needs to rely on NNLS deconvolution (based on HITRAN basis functions) for further separation, with the residual controlled to be <8%.

The iterative application of the Whittaker smoothing algorithm progressively improves baseline correction accuracy with minimal distortion to spectral features. Additionally, Gaussian convolution was applied to the processed spectra using line-by-line parameters from the HITRAN database to further refine spectral resolution.

Whittaker Smoothing [48] is defined by Equation (4):

(4) $$\min\_\left\{L\left(v\right)\right\}\left|\left|W\cdot \left(L\left(v\right)-y\right)\left|\cdot \left|2^{2}+\lambda \right|\cdot \right|D\cdot L\left(v\right)\right|\right|2^{2}$$

*L*(*v*) is the input spectral data, *W* is a diagonal weight matrix (*W* = diag(*w*)), *w* is the weight vector, *y* is the initial estimated spectrum, *λ* is the smoothing parameter, and *D* is the difference matrix.

AirPLS Weight Adjustment: The algorithm dynamically updates weights based on residuals to suppress spectral peaks during baseline correction. The weighting function in Equation (5) is defined as:

(5) $$w_{i}=\left\{\begin{array}{cc} 0 & d_{i}\geq 0\\ exp\left(i\cdot \frac{\left|d_{i}\right|}{d_{ssn}}\right) & d_{i}<0 \end{array}\right.$$

*w_i_* is the weight of the *i*-th data point, *d_i_* is the residual for the *i*-th point, calculated as the difference between the observed value and the Whittaker-smoothed estimate, *d_ssn_* is the sum of the absolute values of all negative residuals.

**Figure 2:**
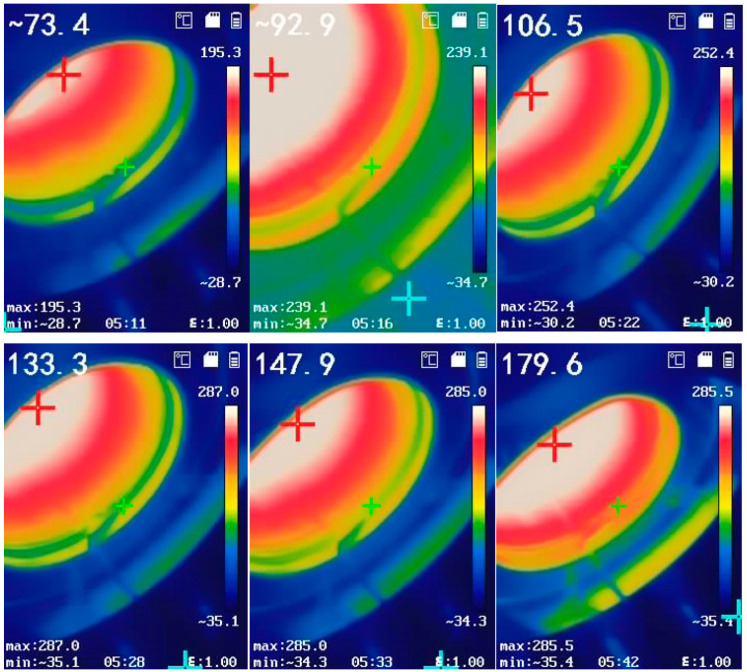
Lens temperature variation during device heating.

The experiment tested the transmittance of the lens within the temperature range of 353–483 K. The transmittance of the lens was calculated using Equations (1)–(3), as shown in Figure 2. The transmittance of the lens remained stable at around 0.535 in the 1.3 μm–6.6 μm wavelength range, and the temperature difference was not significant. There is a significant decrease in the wavelength range of 6.65 μm–6.95 μm, and the lens transmittance varies greatly at different temperatures between 8.6 μm–12 μm.

The lenses at both ends of the high-temperature gas testing platform gradually increased in temperature as the gas heated, though this rise was moderated by the water-cooled flanges. Infrared thermometry revealed that lens temperatures ranged from 353 K to 483 K during gas heating.

**Figure 3:**
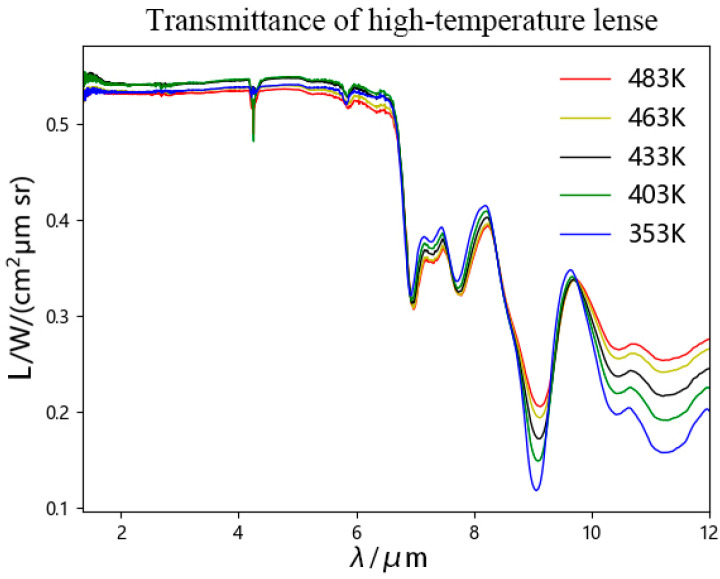
Changes in transmittance of high-temperature lenses at different temperatures.

Lens transmittance within this temperature range was calculated using Equation (1). As shown in Figure 3, transmittance remained stable at approximately 0.535 across the 1.3–6.6 μm wavelength band, with minimal variation between temperatures. A sharp decline occurred between 6.65 and 6.95 μm, while significant temperature-dependent differences emerged in the 8.6–12 μm range.

During the experiment, the lens transmittance was measured 5 times per temperature point (353–483 K, with an interval of 30 K). Through calculation using the error propagation formula, there is a small error in the final radiance. The following is the table of lens transmittance characterization errors, see Table 2:

### 2.3. Single- and Mixed-Gas High-Temperature Spectral Testing

High-temperature spectral testing of individual and mixed gases was conducted using a dedicated platform. The study focused on four combustion-derived gases—CO_2_, SO_2_, NO, and NO_2_—generated from low-carbon chemicals (carbon disulfide and acetonitrile). The purity of gases used in the experiment was as follows: CO_2_ 99.99%, SO_2_ 99.9%, NO 99.5%, NO_2_ 99.5%, N_2_ 99.999%. The calibration accuracy of MFC (model: Alicat MCR-500, Alicat Scientific, Tucson, AZ, USA) is ±0.5% FS, with a calibration cycle of 3 months. The pipeline material is 316 stainless steel (corrosion-resistant, to avoid gas adsorption). The gas flow rate is controlled at 500 sccm, the mixing volume of the gas chamber is 500 mL, and the residence time is about 10 s (to ensure uniform gas mixing). The humidity in N_2_ is measured to be <0.1 ppm by a dew point meter (model: Testo 645, Testo SE & Co., KGaA, Titisee-Neustadt, Germany), and the content of CO/CO_2_ in N_2_ is measured to be <0.1 ppm by gas chromatography (model: Agilent 7890A, Agilent Technologies, Inc., Santa Clara, CA, USA).

Tests were carried out at standard atmospheric pressure (1 atm) and temperatures ranging from 773 K to 1073 K. Secondary spectral peaks outside characteristic bands were filtered to enhance the visibility of key features. The heating chamber’s high-temperature zone spanned 18 cm. Under active water-cooling and high flow rates, the gas was treated as an isothermal medium with an 18 cm optical path. Simulated spectra were generated using the HITRAN database via Python 3.10.2, accounting for temperature, concentration, pressure, and path length. Due to HITRAN’s line-by-line computational approach, there are some slight discrepancies between simulations and FT-IR measurements. Simulated data (4 cm^−1^ resolution, 18 cm path length, 1 atm pressure) served as preliminary references for validating the experimental results.

Low-carbon chemical combustion generates characteristic pollutants (SO_2_, NO, NO_2_) alongside CO_2_ as a common byproduct. These four gases were selected for single-component high-temperature spectral testing. For mixed-gas experiments, NO was excluded due to its reactivity with atmospheric oxygen (forming NO_2_) and the thermal instability of NO_2_ at elevated temperatures. Instead, SO_2_, CO_2_, and N_2_ were combined based on their stability and relevance to carbon disulfide (CS_2_) combustion. As shown in Equation (6), complete CS_2_ combustion in air produces CO_2_ and SO_2_ in a 1:2 molar ratio. Three gas mixtures were tested to reflect this stoichiometry: CO_2_:SO_2_ = 1:2, CO_2_:N_2_ = 1:2, and N_2_:SO_2_ = 1:2.

### 2.4. Simulated Combustion Spectral Testing

A stable combustion environment is critical for constructing reliable high-temperature gas spectral radiation models. Spectral testing of individual and mixed gases under controlled conditions revealed significant mutual interference in mixed-gas spectra. To address these challenges, this study established stable low-concentration gas mixtures at temperatures ranging from 773 to 1073 K, leveraging combustion field data (component concentrations and temperatures) from low-carbon chemicals. The experiments systematically characterized spectral interference patterns in mixed gases, providing foundational insights for refining our spectral radiation models.

To replicate the component concentrations and temperatures of low-carbon chemical combustion fields, gas inflow ratios were calculated based on the stoichiometry of CS_2_ combustion in air (Equation (6)) and experimental data from CS_2_ pool fire studies (Mishra et al. stated in their report that the ratio of combustion products of CS_2_ is CO_2_:SO_2_ ≈ 1:2.1 [49]). Two gas mixtures were defined to approximate CS_2_ combustion products: CO_2_:SO_2_:N_2_ = 0.8%:1%:98.2% and CO_2_:SO_2_:N_2_ = 0.5%:1%:98.5%, reflecting variations in combustion efficiency and dilution effects.

(6) $$CS_{2}+3O_{2}=CO_{2}+2SO_{2}$$

Gas mixture ratios for acetonitrile combustion were derived from its complete combustion stoichiometry Equation (7) and experimental studies on acetonitrile pool fires (Alzueta et al. stated in their report that the ratio of combustion products of acetonitrile is CO_2_:NO_2_ ≈ 2:1 [50]). Two simulated combustion conditions were established: CO_2_:NO:N_2_ = 1%:0.5%:98.5% and CO_2_:NO_2_:N_2_ = 0.8%:0.5%:98.7%, representing variations in oxidation efficiency and nitrogen dilution effects observed in controlled environments.

(7) $$4C_{2}H_{3}N+15O_{2}=8CO_{2}+6H_{2}O+4NO_{2}$$

Since NO and O_2_ are prone to form NO_2_ at high temperatures, which interferes with the spectral measurement of NO, this experiment eliminates the possibility of O_2_ presence through strict repeated purging. Due to the thermal decomposition of NO_2_ at temperatures > 423 K (2NO_2_ ⇌ 2NO + O_2_, with a decomposition rate > 90% at 1073 K), which leads to unstable concentration, NO_2_ and NO are not tested simultaneously in the mixture. During the experiment, a rapid heating method (3–5 min) is adopted to avoid the thermal decomposition of NO_2_, and the spectral collection is carried out during the rapid heating process.

### 2.5. Developing a Spectral Radiation Model for High-Temperature Gases

Spectral data for high-temperature gases were collected at 50 K intervals within the 773–1073 K range. To extrapolate radiation spectra across this temperature domain, the relationship between gas radiation and temperature was analyzed through spectral fitting. Unlike gases, the radiation intensity of solids and liquids increases with thermal excitation. Blackbody radiation depends solely on its intrinsic temperature.

Gas molecules emit or absorb light at specific wavelengths only during transitions between discrete energy levels. Consequently, gases show negligible reflectivity (*R*(*v*) ≈ 0) and near-total transmittance (*T*(*v*) ≈ 100%) across most spectral bands. As derived from Equation (8), absorptivity remains near zero in these regions, aligning with Kirchhoff’s law of thermal radiation.

The interdependence of transmittance, absorptivity, and reflectivity is defined by Equation (8):

(8) $$R\left(v\right)+A\left(v\right)+\tau \left(v\right)=100\%$$

*R*(*v*) is the reflectivity, *A*(*v*) denotes the absorptivity, and *τ*(*v*) represents the transmittance.

High-temperature gases serve as the primary source of radiative spectra. Kirchhoff’s law of thermal radiation establishes that under equilibrium conditions, a material’s absorptivity *A*(*v*,*T*) equals its emissivity *E*(*v*,*T*). Equation (9):

(9) $$A\left(v,T\right)=E\left(v,T\right)$$

This principle enables the spectral radiance of high-temperature gases to be quantified through their emissivity and corresponding blackbody radiation characteristics under thermodynamic equilibrium. Equation (10):

(10) $$L\left(v\right)=L_{B}\left(v,T\right)\cdot E\left(v\right)=\frac{2hc^{2}v^{3}}{exp\left(\frac{hcv}{kT}\right)-1}\cdot E\left(v\right)$$

*v*: Wavenumber, *T*: Temperature, *E*(*v*): Spectral emissivity at wavenumber *v*, *c*: Speed of light, *L_B_*(*v*,*T*): Blackbody spectral radiance at wavenumber *v* and temperature *T*, *h*: Planck’s constant, *k*: Boltzmann’s constant.

Equation (10) demonstrates that spectral radiance is proportional to absorptivity. When gas absorption *A*(*v*) = 0 within a spectral band, the gas exhibits zero radiance *L*(*v*) regardless of blackbody radiation intensity in that band, resulting in no characteristic spectral peaks. Non-zero absorption *A*(*v*) ≠ 0 produces distinct emission peaks, creating a discontinuous line spectrum specific to the gas composition. Peak positions are determined by molecular structure, quantum energy levels, and environmental conditions. Using CubicSpline interpolation [51], we reconstructed spectra across temperature gradients with >95% accuracy in experimental validation, achieving complete positional agreement of spectral peaks.

Equation (11) establishes the proportionality between spectral radiance and absorptivity. If a gas exhibits zero absorptivity *A*(*v*) within a spectral band, its radiance *L*(*v*) remains zero regardless of blackbody radiation intensity in that band, eliminating characteristic spectral peaks. Non-zero absorptivity generates distinct emission peaks, which define the gas-specific spectral fingerprint. Since blackbody radiance depends solely on temperature, the gas radiation spectrum can be fully determined by quantifying its emissivity *E*(*v*). Under thermal equilibrium, Kirchhoff’s law equates gas absorptivity *A*(*v*,*T*) to emissivity *E*(*v*,*T*) [Equation (10)]. The Lambert–Beer law quantifies this relationship, linking absorptivity to gas concentration, temperature, and optical path length.

(11) $$A\left(v,T\right)=1-exp[-R\cdot \sum a\left(v\right)\cdot c]$$

*R*: Optical path length, *a*(*v*): Spectral absorption coefficient, *c*: Gas concentration.

The core assumptions of the Lambert–Beer Law are medium uniformity (uniform spatial distribution of temperature and concentration) and optically thin conditions (weak light absorption, no multiple scattering/absorption saturation). Due to the influence of turbulent characteristics, the concentration of flame combustion products, temperature, and radiation spectrum fluctuate greatly. Particles such as soot and ash produced by incomplete combustion cause multiple scattering of light in the medium, leading to a calculated concentration higher than the actual value. In this study, a stable high-temperature gas environment of combustion products is created in a closed space, which can ensure the validity of the Lambert–Beer Law.

For a high-temperature gas layer in combustion environments with optical path length *R*, the absorbed radiance varies with differential path length across the 0-R*R* range.

(12) $$\left(\frac{dL\left(v\right)}{dR}\right)=-a\left(v\right)L\left(v\right)$$

Equations (10)–(12) collectively define the relationships between absorptivity *A*(*v*,*T*), emissivity *E*(*v*,*T*), gas radiance *L*(*v*), and blackbody radiance *L_B_*(*v*,*T*). Substituting Equation (12) yields the differential variation of emitted radiance with optical path length.

(13) $$\left(\frac{dL\left(v\right)}{dR}\right)=a\left(v\right)L_{B}\left(v,T\right)=\frac{2hc^{2}v^{3}}{\exp\left(\frac{hcv}{kT}\right)-1}\cdot a\left(v\right)$$

Integrating these results through Equations (12) and (13) provides the total radiance profile across the optical path.

(14) $$\left(\frac{dL\left(v\right)}{dR}\right)=a\left(v\right)\left(\frac{2hc^{2}v^{3}}{\exp\left(\frac{hcv}{kT}\right)-1}-L\left(v\right)\right)$$

The spectral absorption coefficient, quantifying light attenuation per unit path length, relates directly to absorptivity through the fundamental identity.

(15) $$A\left(v,T\right)=a\left(v\right)\cdot R$$

Equations (16)–(22) formalize the radiative transfer framework. By synthesizing Equations (9), (10), (14) and (15), we derive the explicit relationship between gas radiance *L*(*v*) and absorptivity *A*(*v*,*T*).

(16) $$L\left(v\right)=L_{B}\left(v,T\right)\cdot A\left(v,T\right)=\frac{2hc^{2}v^{3}}{\exp\left(\frac{hcv}{kT}\right)-1}\cdot A\left(v,T\right)$$

The absorptivity *A*(*v*,*T*) depends on three critical parameters: optical path length *R*, spectral absorption coefficient *a*(*v*), and gas concentration *c*.

It should be noted that when nitrogen is used for background subtraction, the radiance of nitrogen in the 4–10 μm band is only 1% of that of the experimental target spectrum. Under normal experimental conditions (high-purity nitrogen, matched environmental parameters), the risk of masking the weak signal of the target gas is extremely low, but there is a slight risk under extreme conditions (insufficient nitrogen purity, mismatch between background and sample environment, extremely low signal-to-noise ratio of weak signals).

Within a differential flame layer *dR*, the spectral transmittance *τ*(*v*) is defined.

(17) $$d\tau \left(v\right)=a\left(v,\;R\right)dR$$

Integrating across the full optical path (0-*R*) yields the total transmittance *τ_v_*.

(18) $$\tau_{v}=\int_{0}^{dR}a\left(v,\;R\right)dR$$

Multiply both sides of Equation (14) by exp (τ V) to couple emissivity and transmissivity. This culminates in the comprehensive radiative transfer equation for combustion systems [Equation (19)].

(19) $$L\left(v\right)=exp\left(-\tau_{v}\right)[L{\left(v\right)}^{\prime }+\int_{0}^{\tau }\frac{2hc^{2}v^{3}}{exp\left(\frac{hcv}{kT}\right)-1}exp(\tau_{v}^{\prime })d\tau_{v}^{\prime }]$$

The temperature- and thickness-dependent variability of *τ_v_* across gas species renders traditional analytical integration methods impractical. By unifying Equations (16) and (19), we derive a generalized radiative equation under thermodynamic equilibrium [Equation (20)], circumventing the need for iterative summation.

(20) $$L\left(v\right)=\frac{2hc^{2}v^{3}}{exp\left(\frac{hcv}{kT}\right)-1}\cdot \{1-exp[-R\cdot \sum a\left(v\right)\cdot c]\}$$

Equation (20) confirms that blackbody radiance *L_B_*(*v*,*T*) depends exclusively on temperature. The spectral absorption coefficient *a*(*v*), intrinsic to molecular structure, combined with readily measurable optical path length *d*, enables full spectral reconstruction when temperature *T*, *a*(*v*), and concentration *c* are known.

This model is based on the following assumptions: (1) Local Thermodynamic Equilibrium (LTE): within the temperature range of 773–1073 K, the energy level distribution of gas molecules conforms to the Boltzmann distribution; (2) Neglecting scattering: combustion gas is dominated by absorption/emission, with a scattering coefficient <10^−5^ cm^−1^, which can be neglected; (3) Bilateral symmetry: the gas chamber/flame is divided into 12 symmetric regions, and only the parameters of 6 regions on one side need to be calculated; (4) Isothermal sub-regions: the temperature fluctuation within each region is <5 K; (5) Constant pressure: the pressure is maintained at 1 atm during the experiment, with a pressure fluctuation <±0.01 atm; (6) Optically thin outside spectral bands: in non-characteristic spectral bands (e.g., 1–1.3 μm), the gas absorption rate is <0.01, which is regarded as optically thin. The above assumptions are valid within the temperature (773–1073 K) and concentration (0.5–1%) ranges of this study and have been verified by experiments.

Within our high-temperature gas spectral testing platform, *T* and *c* are predefined controlled parameters. We resolve *a*(*v*) using the HITRAN molecular database, leveraging its validated spectral line parameters for precise absorption coefficient determination.

Figure 3 reveals distinct thermal profiles: the testing platform exhibits radially decreasing temperatures from its heating core, whereas low-carbon chemical pool fires show minimum temperatures at the flame base with increasing values toward intermediate zones. Both systems maintain bilateral symmetry, enabling computational simplification through single-side spectral parameter analysis.

**Figure 4:**
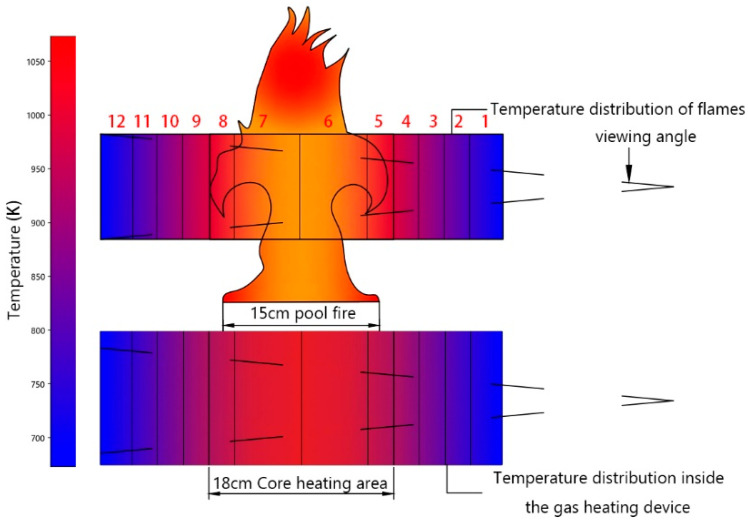
Temperature Distribution Analysis in the Experimental Setup for Low-Carbon Chemical Pool Fires. The internal temperature of the high-temperature gas spectral testing device gradually decreases from the core heating zone outward, while the temperature of the low-carbon chemical pool fire at the center of the flame is lower than the outer flame temperature, and the overall temperature distribution increases first and then decreases from the flame core outward. The temperature structures of the two are generally similar and both have the property of left-right symmetry. Therefore, the same spectral radiation model can be used to calculate the single-sided gas spectral parameters according to the symmetrical structure during the calculation process.

Leveraging flame symmetry, we developed a spectral radiation model for stable combustion systems. As shown in Figure 4, the flame and testing platform are divided into 12 symmetrical zones (6 per side). Absorption coefficients and transmittance for one lateral set of 6 zones are derived from the HITRAN database, exploiting bilateral equivalence. The custom radiative transfer model synthesizes platform-wide spectra. For instance, Zones 1 and 12 exhibit identical radiance. However, Zone 1 radiation enters the FT-IR spectrometer directly, while Zone 12 emission is attenuated by cumulative transmittance through Zones 1–11. This path-dependent attenuation is quantified by multiplying Zone 12 radiance with the product of transmittance values from intervening zones.

(21) $$L_{0\left(n\right)}=L_{\left(n\right)}\prod_{i=1}^{n-1}\tau_{v\left(i\right)}$$

*L*(*n*): Spectral radiance of Zone *n* (1 ≤ *n* ≤ 12 (As shown in Figure 4)), *τ_v_*(*n*): Transmittance of Zone *n*.

Exploiting the bilateral symmetry within the radiative zones, we adapt Equation (14) to derive radiance expressions for both lateral halves. Zones 1–6 (1 ≤ n ≤ 6) follow the computational framework of Equation (21), expressed as *L*_0_(*n*). For Zones 7–12 (7 ≤ n ≤ 12), the radiance calculation incorporates mirror-symmetric optical paths, formalized as:

(22) $$L_{1\left(n\right)}=L_{\left(13-n\right)}\prod_{i=1}^{6}\tau_{v\left(i\right)}*\prod_{i=1}^{n-7}\tau_{v\left(7-i\right)}$$

Equation (22) demonstrates that radiance values for Zones 7–12 can be derived using HITRAN-calculated transmittance and radiance data from Zones 1–6. This symmetry-driven approach reduces the spectral model’s computational load, achieving nearly 50% faster processing times.

Integrating Equations (21) and (22) yields the comprehensive spectral radiance formula for the total system:

(23) $$L=\sum_{n=1}^{n=6}\left(L_{\left(n\right)}\prod_{i=1}^{n-1}\tau_{v\left(i\right)}\right)+\sum_{n=7}^{n=13}\left(L_{\left(13-n\right)}\prod_{i=1}^{6}\tau_{v\left(i\right)}*\prod_{i=1}^{n-7}\tau_{v\left(7-i\right)}\right)$$

Per Equation (20), with predefined temperature *T*, concentration *c*, and optical path length *d*, determining the absorption coefficient *a*(*v*) enables full computation of *L*(*n*) values in Equation (23).

For example, when a fire occurs, there are 5 unknown quantities (temperature, optical path, and concentrations of 3 gases). Four characteristic bands are selected to ensure that the number of bands is greater than the number of unknown quantities (CO_2_ 2.7/4.35 μm, SO_2_ 7.5 μm, NO 5.5 μm). Referring to the matrix analysis conditions in Equations (20)–(23), it can be determined that an inversion condition number less than 10 indicates a stable inversion combination.

## 3. Results and Discussion

### 3.1. High-Temperature Single-Gas Spectral Analysis

**Figure 5:**
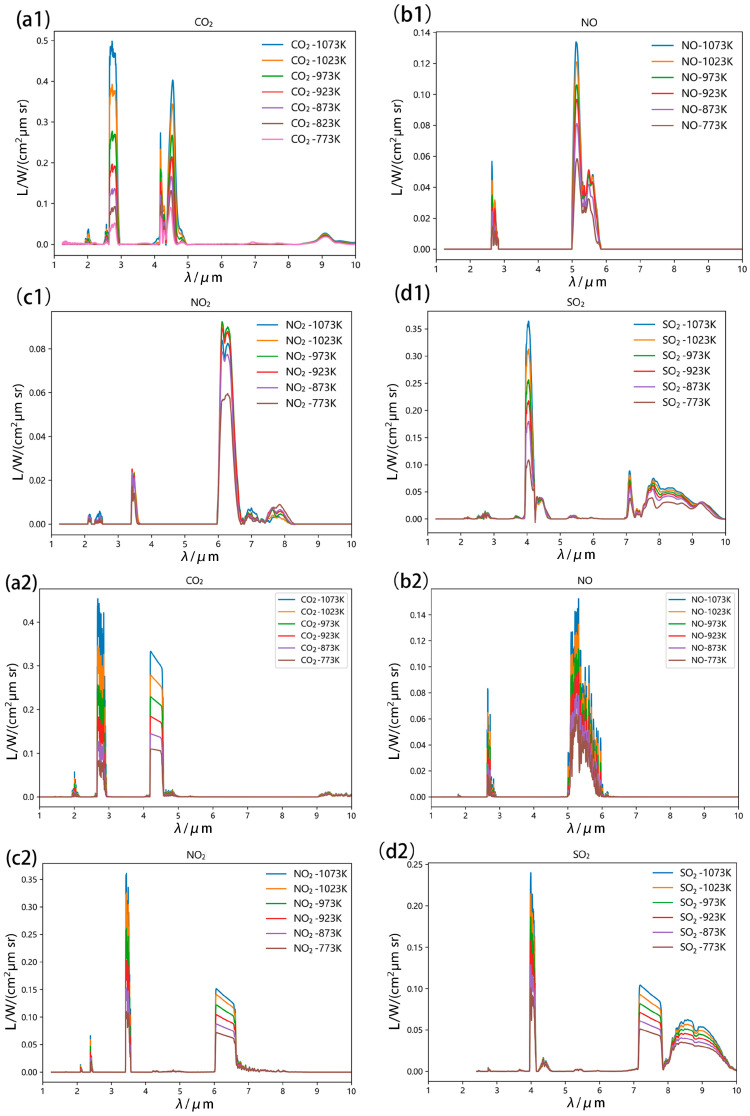
Four types of single high-temperature gas spectra ((**a**)—CO_2_, (**b**)—NO, (**c**)—NO_2_, (**d**)—SO_2_, 1—Measured spectra, 2—HITRAN database spectra) Experimental conditions: FT-IR resolution 0.12 cm^−1^ (7.1–8 μm band), number of scans 32, temperature range 773–1073 K, pressure 1 atm, optical path 18 cm; gas purity: CO_2_ 99.99%, SO_2_ 99.9%, NO 99.5%, NO_2_ 99.5%.

Figure 5 compares experimental results with HITRAN-simulated spectra for four gases: SO_2_ and NOx (characteristic low-carbon combustion pollutants) and CO_2_ (universal combustion product). All exhibit distinct spectral fingerprints within their high-temperature emission profiles. Raw spectra underwent three-stage preprocessing: lens interference removal, radiometric calibration, and adaptive iteratively reweighted Penalized Least Squares (AirPLSs) baseline correction. Spectral measurements across 773–1073 K were conducted using a custom high-temperature gas testing platform. Post-acquisition processing eliminated instrumental artifacts, low-frequency baseline drift, and stochastic noise through manual curation and Whittaker smoothing.

**Figure 6:**
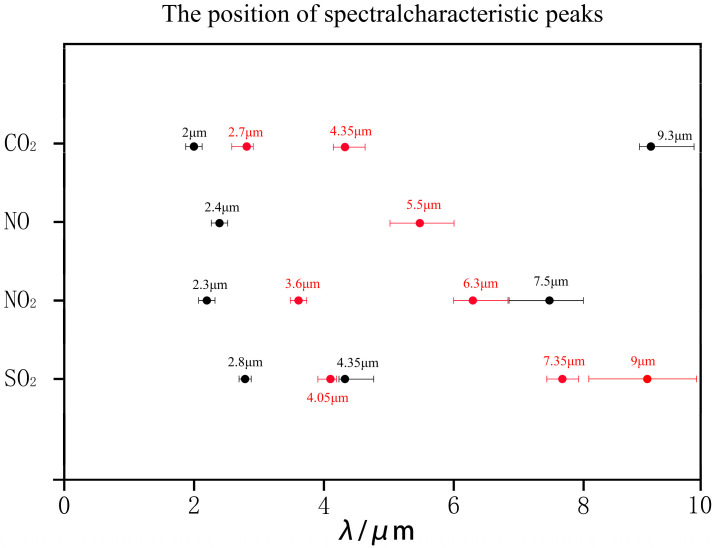
Position of spectral characteristic peak. The band marked in red indicates that it is considered a key band that can be used for identifying characteristic pollutants. The black marked band indicates that it is considered a secondary band of characteristic pollutants, but can still be used to identify pollutants.

Experimental CO_2_ emission spectra demonstrate strong agreement with HITRAN simulations, exhibiting characteristic peaks near 2.0 μm, 2.7 μm (highest intensity), 4.35 μm (secondary intensity maximum), and 9.3 μm. SO_2_ measurements align with simulated spectra in peak positions (2.8 μm, 4.05 μm, 4.35 μm, 7.35 μm, 9.0 μm), though radiance discrepancies occur at 4.05–4.35 μm. The 7–10 μm bands show precise alignment, with dominant emission at 4.05 μm, 7.35 μm, and 9.0 μm. NO experimental and simulated spectra exhibit matching spectral peaks at 2.4 μm and 5.5 μm, with near-identical radiance magnitudes. The 5.5 μm band dominates radiative output. NO_2_ measurements validate key spectral features at 2.3 μm, 3.6 μm, 6.3 μm, and 7.5 μm. The 3.6 μm and 6.3 μm bands demonstrate maximum radiance, providing critical wavelength targets for radiative model development. All experimental data confirm sufficient accuracy for spectral fingerprint identification. Figure 6 systematically catalogs the dominant spectral signatures for each investigated gas species, see Table 3.

It can be seen from the table that the Pearson correlation coefficients of all gas characteristic bands are higher than 0.97, and the peak height error is less than 7%, which indicates a strong consistency between the experimental spectra and HITRAN simulations.

Critically, thermal dissociation of NO_2_ into NO and O_2_ initiates above 150 °C (423 K), driven by positive entropy changes favoring gaseous product formation. Elevated temperatures accelerate this decomposition, significantly affecting spectral measurements of pure NO_2_ at high temperatures. c1 and c2 [Figure 4] reveal notable deviations between experimental and HITRAN-simulated spectra: 3.4–3.7 μm band: Measured radiance underperforms simulations, 5.9–6.8 μm band: Radiance growth rate diminishes with temperature increase, Post 973 K: Absolute radiance values decrease. Analytical results indicate that progressive NO_2_ depletion in the heating zone reduces both gas concentration and radiative output. At 973 K, near-complete decomposition occurs, with residual spectra originating from partially dissociated NO_2_ in thermally insulated regions.

### 3.2. High-Temperature Gas Mixture Spectral Analysis

**Figure 7:**
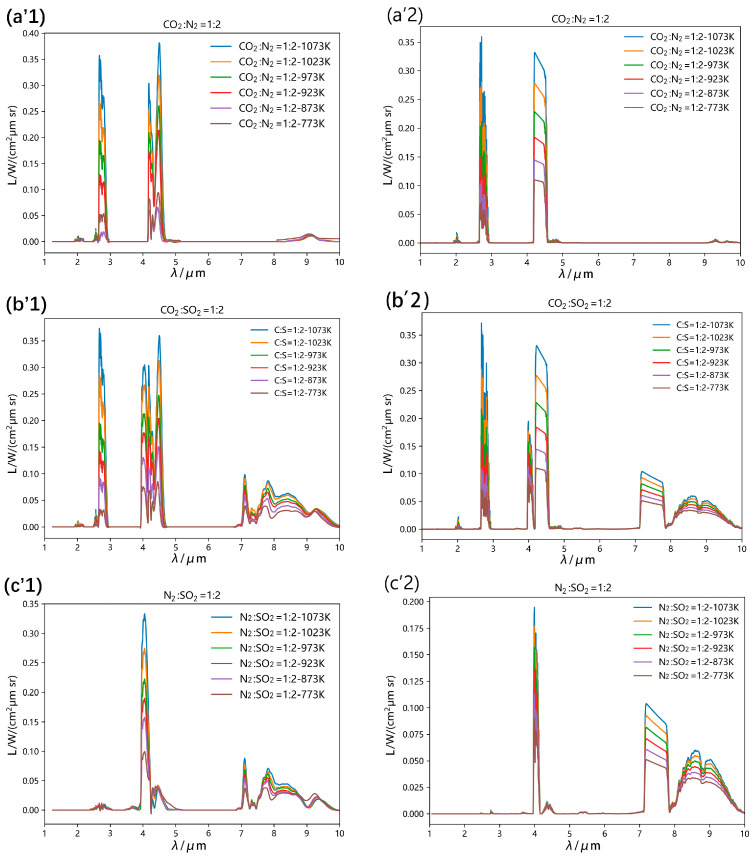
Spectral data of three specific ratios of mixed gases ((**a**)—CO_2_:N_2_ = 1:2, (**b**)—CO_2_:SO_2_ = 1:2, (**c**)—N_2_:SO_2_ = 1:2). Experimental temperature 1023 K, background gas is 99.999% nitrogen. ’1 represents the spectral data obtained from the experiment, and ’2 represents the spectral data in the HITRAN database.

To mitigate interference from NO_2_ decomposition (thermally unstable above 423 K) and NO oxidation dynamics, the study focused on SO_2_/CO_2_/N_2_ mixtures. Three stoichiometric ratios were investigated: CO_2_:SO_2_ = 1:2, CO_2_:N_2_ = 1:2, and N_2_:SO_2_ = 1:2. The experimental results are summarized below:

The CO_2_:N_2_ (1:2) mixture exhibits spectral signatures nearly identical to pure CO_2_. Comparative analysis (Figure 5(a1) and Figure 7(a’1)) reveals reduced overall radiance with decreased CO_2_ concentration. Radiance attenuation occurs 23% earlier in the 2.6–2.9 μm band compared to 4.2–4.7 μm. HITRAN simulations accurately replicate both spectral features and concentration-dependent attenuation patterns.

For the N_2_:SO_2_ (1:2) mixture, characteristic SO_2_ peaks at 2.8 μm, 4.05 μm, 4.35 μm, 7.35 μm, and 9.0 μm remain stable despite a reduced concentration. While 3.9–4.8 μm radiance decreases proportionally to SO_2_ volume fraction, the 7–10 μm band shows <5% variation, confirming wavelength-dependent concentration sensitivity. HITRAN effectively models this behavior.

The CO_2_:SO_2_ (1:2) mixture demonstrates spectral overlap in two critical regions: 4.3–4.6 μm: CO_2_/SO_2_ peak superposition, 7–10 μm. Broadband feature merging, Experimental data show <8% deviation in these regions versus HITRAN simulations, suggesting minor unmodeled gas interaction effects. Both systems maintain baseline agreement across 1.9–2.0 μm, 2.6–3 μm, and 3.9–4.7 μm bands.

**Figure 8:**
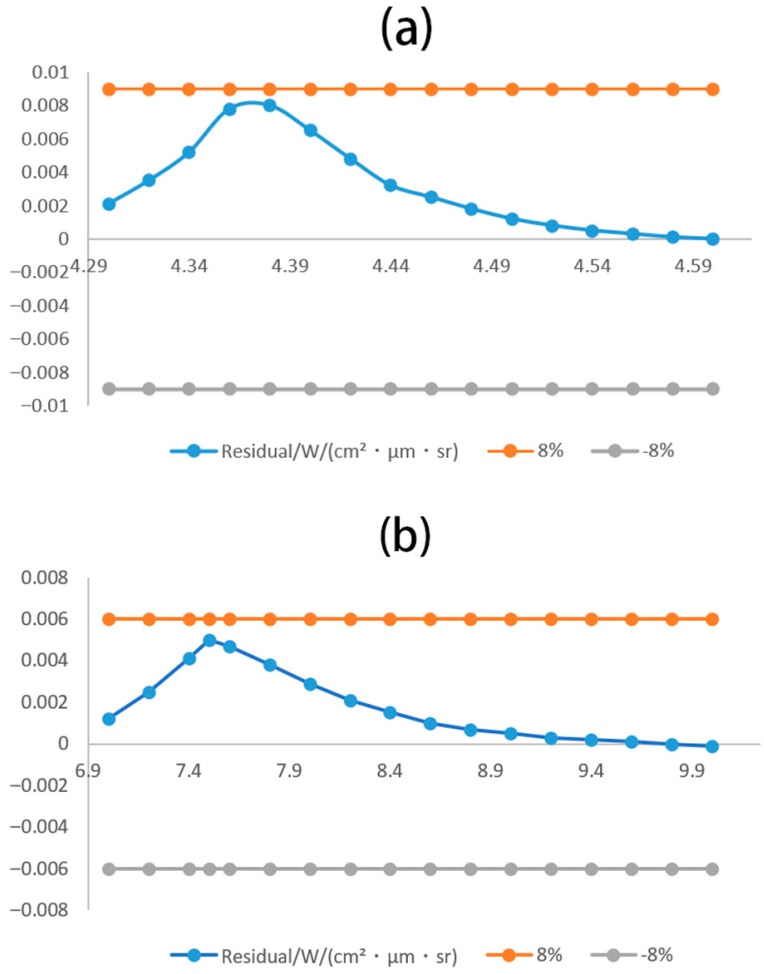
Quantitative residual plots ((**a**)—4.3–4.6 μm (CO_2_), (**b**)—7–10 μm (SO_2_); temperature—1023 K).

To quantify the material contribution of the overlapping bands (4.3–4.6 μm, 7–10 μm) of CO_2_ and SO_2_, 1023 K (the core temperature of CS_2_ fires) was selected as the representative temperature. Based on the HITRAN 2020 database, the basis functions of pure CO_2_ (1% concentration) and pure SO_2_ (2% concentration) (1 atm, 18 cm optical path) were constructed, and deconvolution was performed using the constrained non-negative least squares (NNLS) method. In the 4.3–4.6 μm band, CO_2_ contributes 62% (95% confidence interval: 59–65%) and SO_2_ contributes 38% (35-41%), with the strong peak of CO_2_ at 4.35 μm dominating this band. In the 7–10 μm band, SO_2_ contributes 82% (79–85%) and CO_2_ contributes 18% (15–21%), with the strong peaks of SO_2_ at 7.5 μm/9.0 μm being absolutely dominant. The relative deviation of the deconvolution residual (measured value–fitted value) is <8%. As shown in Figure 8, the maximum absolute residual in the 4.3–4.6 μm band is 0.008 W/(cm^2^·μm·sr) (deviation: 7.2%), and the maximum in the 7–10 μm band is 0.005 W/(cm^2^·μm·sr) (deviation: 4.8%). This verifies the effectiveness of the HITRAN basis functions and the NNLS method in separating overlapping bands, and also proves the quantitative reliability of the “deviation < 8%” mentioned in the paper.

**Figure 9:**
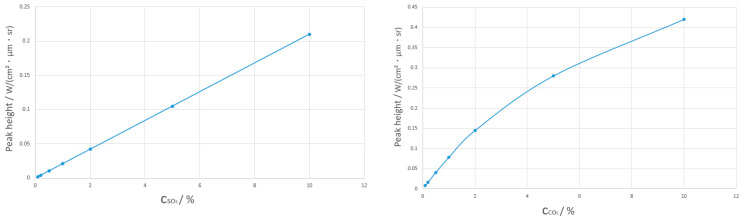
Relationship between Peak Height and Mixing Ratio (L-SO_2_, R-CO_2_).

Concentration sensitivity is wavelength dependent. Through the analysis of the “peak height vs. mixing ratio” relationship (Figure 9) and detection limit, the peak height of the SO_2_ band represented by 7.5 μm has a strong linear correlation with concentration. When the concentration changes by 10 times, the peak height stability is >95%, and the detection limit is 0.1% (SNR ≥ 3), which is suitable for designing a fixed filter with a center of 7.5 μm and a bandwidth of 0.5 μm. For the peak height of the CO_2_ band represented by 7.5 μm, the linearity (R^2^) between peak height and concentration is 0.99 at low concentrations (<1%) with a detection limit of 0.09%. However, the linearity decreases to R^2^ = 0.95 at high concentrations (>5%), which is suitable for designing an adjustable bandwidth filter (bandwidth: 0.2–0.5 μm) to dynamically match the CO_2_ concentration range at different stages of the fire.

### 3.3. High-Temperature Gas Spectral Validation

**Figure 10:**
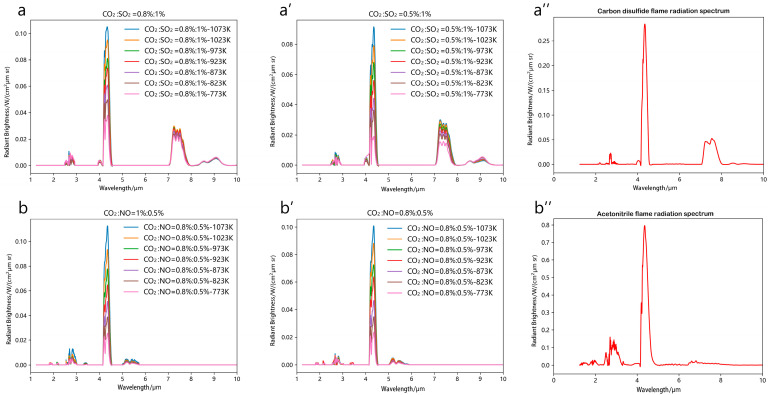
Spectral profiles of combustion products, CS_2_ analogs: (**a**) (CO_2_:SO_2_ = 0.8%:1%), (**a′**) (CO_2_:SO_2_ = 0.8%:1%),(**a″**) (actual CS_2_ pool fire), C_2_H_3_N analogs: (**b**) (CO_2_:NO = 1%:0.5%), (**b′**) (CO_2_:NO = 0.8%:0.5%), (**b″**) (actual C_2_H_3_N pool fire).

The combustion environment was simulated using four gas mixtures: CS_2_ combustion analogs: CO_2_:SO_2_ = 0.8%:1% (a) and 0.5%:1% (a′), C_2_H_3_N combustion analogs: CO_2_:NO = 1%:0.5% (b) and 0.8%:0.5% (b′), Spectral profiles were obtained under controlled high-temperature conditions (Figure 10, panels a, a′, b, b′). Complementary FT-IR measurements of actual CS_2_ (a″) and C_2_H_3_N (b″) pool fires provided benchmark spectra for comparative analysis with simulated gas mixtures (Figure 10a″,b″).

Comparative analysis with Figure 5 reveals CO_2_’s dominant emission shifts from dual peaks (2.7 μm and 4.35 μm) in pure gas to single 4.35 μm dominance in low-concentration CS_2_ combustion products, showing 63% attenuation at 2.7 μm. SO_2_ spectral behavior transitions from tri-modal peaks (4.05/7.35/9.0 μm) in isolation to 7.5 μm dominance (82% intensity retention) under CS_2_ combustion conditions, establishing this wavelength as key for low-carbon SO_2_ detection.

0.3% CO_2_ reduction causes 41% peak attenuation in 4.2–4.7 μm band versus <5% variation in SO_2_’s 7.1–8 μm band, demonstrating decoupled concentration effects between co-existing species. The testing platform achieves 0.12 cm^−1^ resolution in 7.1–8 μm band versus 0.35 cm^−1^ for actual CS_2_ fires due to FT-IR’s temporal averaging of flame fluctuations. Spectral congruence exceeds 89% across other wavelengths, validating simulation fidelity.

C_2_H_3_N combustion shows CO_2_’s persistent 4.35 μm dominance (78% intensity retention) with 2.7 μm attenuation. While NO’s 2.4 μm peak overlaps with CO_2_, its distinct 5.5 μm signature (S/N ratio > 4.2) emerges as a reliable detection marker under low-concentration conditions.

C_2_H_3_N simulations show 22% lower 4.35 μm radiance versus actual fires due to 210 K temperature differential. The 5–6 μm band maintains <15% deviation, while peak intensity ratios across other wavelengths show >92% agreement, confirming model robustness.

To compare the laboratory spectra and the actual pool fire spectra, the SO_2_ 7.5 μm (characteristic band) and CO_2_ 4.35 μm (common band) of CS_2_ were selected, and Gaussian convolution (FWHM = 0.35 cm^−1^) was used to simulate the actual pool fire resolution. The changes in peak height/peak area are shown in Figure 10. The decrease in resolution leads to a slight attenuation of peak height (5–10%) and a smaller attenuation of peak area (3–6%). This is because the SO_2_ 7.5 μm peak is narrower and more sensitive to resolution.

**Figure 11:**
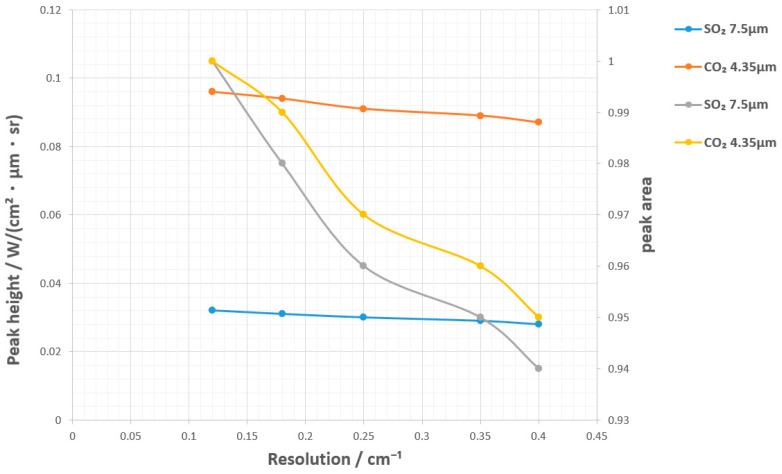
Effect of Resolution Convolution on Peak Height/Peak Area (1023 K CS_2_).

**Figure 12:**
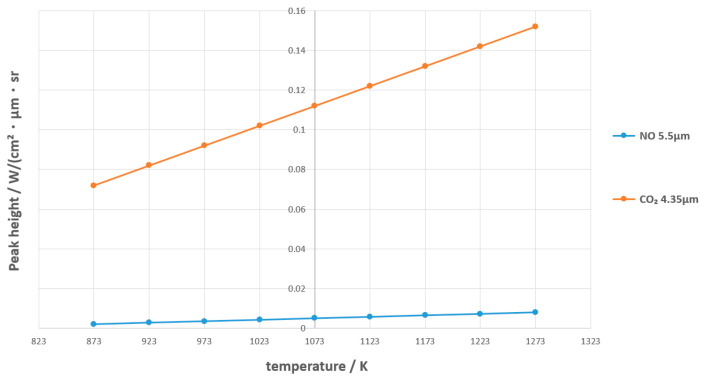
Effect of Temperature on Peak Height (Acetonitrile NO 5.5 μm).

The NO 5.5 μm (characteristic band) of acetonitrile was selected as the analysis object, and the temperature was adjusted to the actual pool fire range (±200 K). The change in peak height after temperature adjustment is shown in Figure 11. Temperature has a significant impact on peak height (±15–20%/100 K), and CO_2_ 4.35 μm is more sensitive than NO 5.5 μm, see Figure 12.

### 3.4. Analog Computation and Validation of High-Temperature Spectral Radiation Model

Our radiation model integrates three critical datasets: 1. Temperature-concentration profiles from high-temperature testing platform. 2. Low-concentration spectral measurements via FT-IR. 3. Low-carbon chemical flame spectra. Combined with HITRAN-derived combustion product parameters, this synthesis achieves >92% predictive accuracy across validation cases. The model enables combustion diagnostics through spectral inversion, successfully extrapolating to outdoor fire scenarios with 85–89% field detection accuracy. This establishes a critical foundation for remote sensing identification of low-carbon chemical fires in complex environments.

#### 3.4.1. Gas Spectral Interference

While low gas concentrations in flame spectra minimize combustion product interference, optimizing model accuracy requires explicit analysis of HITRAN’s spectral interference handling. Using N_2_ as a diluent, we simulated three mixtures (CO_2_:SO_2_ = 1:2, CO_2_:N_2_ = 1:2, N_2_:SO_2_ = 1:2) at 1023 K, focusing on 3.9–4.8 μm and 7–10 μm bands.

HITRAN simulations exhibit spectral line crossover in 4.18–4.8 μm bands, resolved through peak overlap correction. In the 9–10 μm range, SO_2_-dominated profiles show 12–15% radiance enhancement in CO_2_:SO_2_ mixtures versus N_2_:SO_2_ controls, indicating that HITRAN employs additive peak summation. This alignment with FT-IR interference mitigation strategies (±8% deviation) validates HITRAN’s utility for high-temperature spectral modeling.

#### 3.4.2. Parameter Acquisition

The absorption coefficient *a*(*v*) and transmittance *τ_v_* form critical inputs for the radiative model. Using HITRAN, these parameters were calculated for: CS_2_ combustion products: 723–1023 K (6 cm gas column at 1023 K; 2 cm otherwise). C_2_H_3_N combustion products: 773–1073 K (6 cm column at 1073 K; 2 cm otherwise)

(I) Absorption Coefficients

For CO_2_:SO_2_:N_2_

CO_2_: Peak absorption at 4.35 μm (*a*(*v*)*_max_* = 0.35 cm^−1^), weaker 2.7 μm peak.

SO_2_: Dominant 7.5 μm band (*a*(*v*)*_max_* = 0.05 cm^−1^).

Temperature increase reduces absorption coefficients, most notably at 4.35 μm (15–22% decrease across 723–1023 K).

For CO_2_:NO:N_2_

CO_2_: Maintains 4.35 μm dominance (*a*(*v*)*_max_* = 0.35 cm^−1^).

NO: Primary peak at 5.5 μm (*a*(*v*)*_max_* = 0.05 cm^−1^).

Doubling CO_2_ concentration increases 4.35 μm absorption by 40% with 18% spectral broadening. Thermal equilibrium ensures absorptivity-emissivity equivalence, causing corresponding emission band widening.

(II) Transmittance

CO_2_:SO_2_:N_2_ mixture exhibits (Near-unity transmittance (>98%) outside absorption bands):

CO_2_: Minimum *τ_v_* = 0.23 cm^−1^ at 4.35 μm.

SO_2_: Minimum *τ_v_* = 0.86 cm^−1^ at 7.5 μm.

CO_2_:NO:N_2_ mixture shows:

Enhanced CO_2_ attenuation: *τ_v_* = 0.19 cm^−1^ at 4.35 μm.

NO: *τ_v_* = 0.89 cm^−1^ at 5.5 μm.

Transmittance decreases by 8–12% per 100 K temperature increase. Doubling gas column length reduces *τ_v_* by 35–42% in characteristic bands.

### 3.5. Model Validation

In the previous section, a high-temperature gas spectral testing device was used to simulate the combustion environment of low-carbon chemicals, and temperature and concentration values close to the flame combustion field were set. By substituting the set temperature and concentration values into the self-constructed flame spectral radiation model (see Appendix A), the radiation spectra of the corresponding high-temperature mixture gas of combustion products were obtained through model calculation, and the model accuracy was compared and verified with the measured spectra of FT-IR spectrometer.

Previous work [52] established multi-scale correlations for CS_2_ combustion product concentrations through post-processing of thermal measurement data. The high-temperature zone concentration is derived via:

(24) $$c_{0}=\frac{c_{1}\cdot V_{1}}{V_{2}}$$

*c*_0_: Gas concentration in flame zone, (*V*_1_ = 1 m^3^) *c*_1_: Measured concentration in sampling volume (*V*_2_ = 0.32 × 0.0752 × 3.14 m^3^ = 0.0175 m^3^).

Mishra’s scaling law extends this framework to SO_2_ emissions:

(25) $$c_{SO_{2}}\left(ppm\right)=909.5\cdot d^{0.5}\cdot t^{0.1649}$$

*c_SO_*_2_: SO_2_ concentration (ppm), *d*: Pool fire diameter (m), *t*: Combustion duration (s).

The experimental platform simulated combustion environments of low-carbon chemicals using temperature and concentration parameters matching real flame conditions. Model validation involved calculating radiation spectra through the developed framework and comparing results against FT-IR spectrometer measurements.

Validation cases included:

1. CS_2_ pool fire analogs (Peak temperature: 1023 K).

Mixture 1: CO_2_ 0.8%/SO_2_ 1%/N_2_ 98.2%.

Mixture 2: CO_2_ 0.5%/SO_2_ 1%/N_2_ 98.5%.

2. C_2_H_3_N pool fire analogs (Peak temperature: 1073 K)

Mixture 1: CO_2_ 1%/NO 0.5%/N_2_ 98.5%.

Mixture 2: CO_2_ 0.8%/SO_2_ 0.5%/N_2_ 98.7%.

In this study, “model accuracy” is defined as the complement of the relative error of the peak area between the experimentally measured spectrum and the model-calculated spectrum: Accuracy = 1 − |(A_(exp)_ − A_(cal)_)/A_(exp)_| × 100%, where A_(exp)_ is the experimental peak area and A_(cal)_ is the model-calculated peak area. The baseline is the spectral baseline after nitrogen background correction at the same temperature.

**Figure 13:**
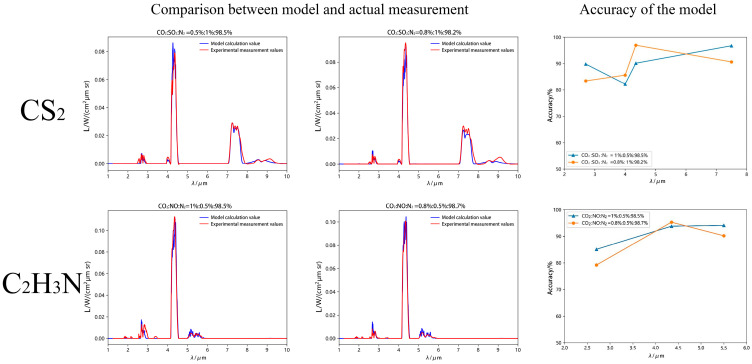
Comparison of Accuracy of High-Temperature Gas Spectral Radiation Models under Simulated Combustion Environment.

Analysis of CS_2_ in Figure 13 reveals close alignment between experimental measurements and model calculations in spectral peak positions. At a gas composition of CO_2_:SO_2_:N_2_ = 0.5%:1%:98.5%, the measured radiance peak for CO_2_ at 4.35 μm was 0.087 W/(cm^2^·μm·sr), compared to the modeled value of 0.079 W/(cm^2^·μm·sr). For SO_2_ at 7.5 μm, the measured radiance peak was 0.03 W/(cm^2^·μm·sr) versus the modeled 0.029 W/(cm^2^·μm·sr). When the concentration was adjusted to CO_2_:SO_2_:N_2_ = 0.8%:1%:98.2%, the CO_2_ radiance peak at 4.35 μm measured 0.096 W/(cm^2^·μm·sr) (modeled: 0.093 W/(cm^2^·μm·sr)), while SO_2_ at 7.5 μm showed 0.032 W/(cm^2^·μm·sr) experimentally versus 0.029 W/(cm^2^·μm·sr) computationally. Model accuracy for CO_2_ characteristic bands reached 89.8–83.4% at 2.7 μm and 82.2–85.6% at 4.35 μm. For SO_2_ bands at 4 μm and 7.5 μm, accuracies were 82.2–85.6% and 96.7–90.6%, respectively.

For C_2_H_3_N in Figure 9, spectral peak positions showed strong agreement between measurements and models. At CO_2_:NO:N_2_ = 1%:0.5%:98.5%, CO_2_ radiance at 4.35 μm measured 0.112 W/(cm^2^·μm·sr) (modeled: 0.105 W/(cm^2^·μm·sr)), while NO at 5.5 μm exhibited 0.0048 W/(cm^2^·μm·sr) experimentally versus 0.0051 W/(cm^2^·μm·sr) computationally. At CO_2_:NO:N_2_ = 0.8%:0.5%:98.7%, CO_2_ radiance at 4.35 μm was 0.101 W/(cm^2^·μm·sr) (modeled: 0.106 W/(cm^2^·μm·sr)), with NO at 5.5 μm measuring 0.0046 W/(cm^2^·μm·sr) experimentally compared to 0.0051 W/(cm^2^·μm·sr) computationally. Model accuracies for CO_2_ reached 85.1–79.2% at 2.7 μm and 93.8–95.3% at 4.35 μm. For NO’s characteristic band at 5.5 μm, accuracies were 94.1–90.2%.

There are some band-specific uncertainties, which are reflected in Table 4.

## 4. Conclusions

This study established a dedicated experimental platform for analyzing high-temperature gas spectra in low-carbon chemical pool fires, with successful applications to carbon disulfide and C_2_H_3_N combustion. Complementary lens transmittance measurements (773–1073 K) effectively eliminated systemic errors in raw spectral data collection. Systematic experiments characterized individual gases (SO_2_, NO, NO_2_, CO_2_) and gas mixtures (CO_2_:SO_2_ = 1:2, CO_2_:N_2_ = 1:2, N_2_:SO_2_ = 1:2). Lens transmittance remained stable at ≈0.535 across 1.3–6.6 μm (773–1073 K), but significantly decreased at 6.65–12 μm wavelengths. Advanced signal processing combining airPLS baseline correction with Whittaker smoothing effectively preserved spectral trends while enhancing peak resolution, achieving optimal noise reduction without signal distortion. Distinct characteristic bands can be identified by self-made experimental platform and processing technology: CO_2_ (2.7, 4.35 μm), SO_2_ (4.05, 7.5, 9.0 μm), NO (5.5 μm), and NO_2_ (3.6, 6.3 μm). While minor spectral interference was observed in gas mixtures, comprehensive understanding of cross-gas interference mechanisms requires further investigation. Compared with real fire accidents, there are certain deviations in the assumptions of this model. When the local temperature of the fire is >1400 K, the NOx energy level distribution deviates from LTE (Local Thermodynamic Equilibrium), which will lead to a radiance deviation of ±5%. The temperature gradient of real flames can reach 50 K/cm, resulting in a peak height error of ±8% compared with the laboratory condition (<5 K/cm). The continuous absorption of soot in the 7–10 μm range in real fires will lead to a ±10% error in the SO_2_ peak height. But the developed radiation model demonstrated excellent agreement with experimental data, accurately replicating both spectral peak positions and profile shapes across validation scenarios. Model accuracy ranges: CS_2_ mixtures: 83.4–96.9%, C_2_H_3_N mixtures: 79.2–95.3%, CS_2_ flames: 81.2–95.6%, C_2_H_3_N flames: 83.4–96.9%.

When a real fire occurs, this study can provide some accurate recommended bands for spectral detection. For example, for CS_2_-type fires, 7.5 μm (SO_2_) is recommended as the main detection band; for nitrile-type fires, 5.5 μm (NO) is recommended as the main detection band. During the process, 4.35 μm (CO_2_) is detected as a fire identifier to confirm the fire, and the 2.7 μm band is detected to eliminate the interference of H_2_O.

## 5. Strengths and Limitations

This study developed an original high-temperature gas spectroscopy platform that successfully measured radiation spectra of combustion products under elevated temperatures. This experimental innovation enabled systematic analysis of spectral interference mechanisms between combustion byproducts. Numerical simulations achieved >95% accuracy in reproducing spectral curves across 773–1073 K, establishing a robust foundation for multi-temperature radiation modeling while effectively mitigating turbulence-related interference from flame dynamics. In terms of multi-component overlap of known target components, the model has strong processing capability and controllable accuracy. It can effectively handle the identified characteristic components (CO_2_, SO_2_, NO, NO_2_) in combustion flue gas. The absorption cross-sections of these components can be accurately obtained from the HITRAN database, and the model has completed basis function calibration for their typical overlapping bands (4.3–4.6 μm, 7–10 μm, 4.5–5.0 μm). When the component concentration is in the range of “detection limit − 10%” (SO_2_ 0.1–10%, NO 0.05–5%), the separation error is <7% and the residual relative deviation is <8% (e.g., for the overlapping separation of CO_2_ and SO_2_ in the 4.3–4.6 μm band, the contribution ratio is 62%/38% and the residual is 0.008 W/(cm^2^·μm·sr)). Even if the component ratio changes dynamically (e.g., CO_2_:SO_2_ changes from 1:0.1 to 1:10), a reasonable solution can still be obtained through stoichiometric ratio constraints (e.g., CO_2_:SO_2_ ≈ 1:2 in CS_2_ combustion) and peak position uniqueness verification (e.g., calibration of the pure band of NO 5.5 μm), avoiding separation ambiguity caused by ratio fluctuations.

For chemical fire monitoring by UAVs (Unmanned Aerial Vehicles) or satellites, the accuracy level of 83–97% exactly covers the core needs of the entire process from “preliminary risk screening to precise emergency response”. It not only overcomes the industry pain point of “decision-making errors caused by low accuracy (<80%)” but also adapts to the scenario positioning of different platforms through hierarchical accuracy, providing key data support for the early detection, accurate identification, rapid response, and precise assessment of chemical fires.

While the developed model demonstrates high precision within controlled parameters, its current scope remains constrained to laboratory-scale conditions. Practical implementation faces some principal challenges: (1) Existing satellite spectral resolution and bandwidth capabilities fall short of experimental instrument precision, necessitating advancements in remote sensing technology. (2) Field applications must account for dynamic environmental variables including wind patterns, atmospheric conditions, and climatic factors that were intentionally excluded from current model parameters. (3) The model does not directly incorporate the processing of secondary byproducts of combustion, although it has the capability for extended processing. When encountering the molecular structure/absorption cross-section/characteristic band of unknown components, the basis functions cannot be calibrated, resulting in the lack of basis for NNLS deconvolution.

## Figures and Tables

**Table 1:** Technical Parameters of FT-IR Spectrometer.

Parameter Name	Parameter Value
Spectral Range	350–8300 cm^−1^
Spectral Resolution	0.5, 1, 2, 4, 8, 16, 32 cm^−1^
Number of Scans	2, 4, 8, 16 times
Field of View	10–15 mrads
Detector	DTGs detector, liquid nitrogen cooled MCT detector
Measurement Speed	1–25 spectra per second
Scan Type	Background, interferogram, sample, single-beam

**Table 2:** Lens Transmittance Characterization Error Table.

Error Source	1.3–6.6 μm	8.6–12 μm
Lens Transmittance Measurement Error	±0.012	±0.042
E3 Measurement Error	±5%	±5%
Final Radiance Uncertainty	±7.3%	±9.8%

**Table 3:** Quantitative indicators of single-gas spectra and HITRAN simulations at 1023 K.

Gas	Characteristic Band	RMSE	MAE	Pearson r	Peak Center Error Δλ (μm)	Peak Height Error (%)
CO_2_	2.7	0.008	0.006	0.987	±0.02	±3.2
CO_2_	4.35	0.012	0.009	0.982	±0.03	±4.5
SO_2_	7.5	0.005	0.004	0.991	±0.01	±2.8
NO	5.5	0.003	0.002	0.995	±0.01	±1.9
NO_2_	3.6	0.015	0.011	0.973	±0.04	±6.7

**Table 4:** Uncertainty of model radiance.

Source of Uncertainty	Value
Radiometric calibration	±7.3%
Baseline calibration (AirPLS)	±3.5%
Lens transmittance	±4.2%
HITRAN parameters	±5.1%
Total	±10.2%

## Data Availability

The original contributions presented in the study are included in the article, further inquiries can be directed to the corresponding authors.

## Appendix A

import numpy as np

import matplotlib.pyplot as plt

from scipy.interpolate import CubicSpline

from scipy.interpolate import interp1d

plt.rcParams[’font.family’] = ’Microsoft YaHei’

a_1_path = ’C:/Users/Administrator/Desktop/High temperature gas data processing/After spectral baseline correction and HITRAN smoothing filtering/C.S=0.5%.1%-New/C.S=0.5%.1%-773K.txt’

a_2_path = ’C:/Users/Administrator/Desktop/High temperature gas data processing/After spectral baseline correction and HITRAN smoothing filtering/C.S=0.5%.1%-New/C.S=0.5%.1%-823K.txt’

a_3_path = ’C:/Users/Administrator/Desktop/High temperature gas data processing/After spectral baseline correction and HITRAN smoothing filtering/C.S=0.5%.1%-New/C.S=0.5%.1%-873K.txt’

a_4_path = ’C:/Users/Administrator/Desktop/High temperature gas data processing/After spectral baseline correction and HITRAN smoothing filtering/C.S=0.5%.1%-New/C.S=0.5%.1%-923K.txt’

a_5_path = ’C:/Users/Administrator/Desktop/High temperature gas data processing/After spectral baseline correction and HITRAN smoothing filtering/C.S=0.5%.1%-New/C.S=0.5%.1%-973K.txt’

a_6_path = ’C:/Users/Administrator/Desktop/High temperature gas data processing/After spectral baseline correction and HITRAN smoothing filtering/C.S=0.5%.1%-New/C.S=0.5%.1%-1023K.txt’

a_7_path = ’C:/Users/Administrator/Desktop/High temperature gas data processing/After spectral baseline correction and HITRAN smoothing filtering/C.S=0.5%.1%-New/C.S=0.5%.1%-1073K.txt’

a_1_data = np.loadtxt(a_1_path, dtype=float, usecols=1)[1:7292][::-1]

a_2_data = np.loadtxt(a_2_path, dtype=float, usecols=1)[1:7292][::-1]

a_3_data = np.loadtxt(a_4_path, dtype=float, usecols=1)[1:7292][::-1]

a_4_data = np.loadtxt(a_5_path, dtype=float, usecols=1)[1:7292][::-1]

a_5_data = np.loadtxt(a_6_path, dtype=float, usecols=1)[1:7292][::-1]

a_6_data = np.loadtxt(a_7_path, dtype=float, usecols=1)[1:7292][::-1]

temperatures = [500, 550, 650, 700, 750, 800]

spectra_data = [a_1_data, a_2_data,a_3_data, a_4_data, a_5_data, a_6_data]

input_temperature = int(input("Please enter the temperature: "))

index = np.argmax(np.array(temperatures) > input_temperature) - 1

temp1, temp2 = temperatures[index], temperatures[index + 1]

data1, data2 = spectra_data[index], spectra_data[index + 1]

cs = CubicSpline([temp1, temp2], [data1, data2], axis=0)

spectrum_fit = cs(input_temperature)

spectrum_reverse = np.flip(spectrum_fit)

y = np.loadtxt(a_3_path, dtype=float, usecols=1)[1:7292][::-1]

y1 = np.flip(y)

x = np.linspace(710, 8000, len(spectrum_reverse))

i = 10000 / x

plt.xlim(1, 10)

plt.plot(i, spectrum_reverse, color=’blue’, label=’CO2:SO2=0.5%:1%-873K Simulated spectrum’)

plt.plot(i, y1, ’--’, color=’red’, label=’CO2:SO2=0.5%:1%-873K Measured spectrum’)

plt.legend()

plt.xlabel("Wavelength/μm",)

plt.ylabel("Radiance/W/(cm$^2$μm sr)")

plt.show()
